# Associations of diabetes mellitus with primary open angle glaucoma and Alzheimer’s disease: a large cohort study in UK biobank

**DOI:** 10.3389/fendo.2025.1506560

**Published:** 2025-07-24

**Authors:** Yan Shi, Xinyue He, William Liu, Junming Hu, Wei Qiao Qiu, Xiaoling Zhang, Zhigang Fan

**Affiliations:** ^1^ Beijing Ophthalmology & Visual Sciences Key Laboratory, Beijing Tongren Eye Center Research Ward, Beijing Tongren Hospital, Beijing Institute of Ophthalmology, Capital Medical University, Beijing, China; ^2^ Yale University, New Haven, CT, United States; ^3^ Departments of Medicine (Biomedical Genetics), Boston University School of Medicine, Boston, MA, United States; ^4^ Departments of Psychiatry and Pharmacology, Alzheimer’s Disease Center, Boston University School of Medicine, Boston, MA, United States

**Keywords:** diabetes mellitus, primary open angle glaucoma, Alzheimer’s disease, APOE E4 allele, UK biobank

## Abstract

**Background:**

Recent studies suggest that the diabetes might be associated with higher risk for primary open angle glaucoma (POAG) and Alzheimer’s disease (AD). However, studies have not addressed the critical issue of confounding by indication, and associations have not been evaluated in a large cross-sectional study. We started this cross-sectional study included United Kingdom Biobank (UKBB) participants with complete data (2006-2010) for analysis to explore the associations between diabetes mellitus (DM) and POAG and AD by considering depression and diabetic retinopathy (DR) as intermediate factors.

**Methods:**

28,112 diabetes patients and 471,869 controls without diabetes were included from UKBB. Data on diagnosis of glaucoma, diabetes, depression, Alzheimer’s disease, diabetic retinopathy, apolipoprotein E (*APOE*) E4 genotypes and data from ophthalmologic examinations were gathered. We further collect the prevalence of DM, DR, depression, POAG and AD, gender, *APOE* E4 genotypes, C-reactive protein (CRP) levels to analysis.

**Results:**

Depression, AD, and POAG were more prevalent in participants with DM compared with non-DM participants, and if DM patients had DR, the prevalence of those comorbidities was even higher than those without DR (all p<0.05). DM, DR, AD, and POAG were more prevalent in participants with depression compared with non-depression participants. Specifically, if DM patients had depression, the prevalence of DR and AD were even higher than those without depression (all p<0.05). In addition, using age-adjusted multivariable general linear model (GLM), we found DM and depression were associated with a higher prevalence of POAG in females while DM and *APOE* E4 negative status were associated with a higher prevalence of POAG in males. In both genders, DM, *APOE* E4, and depression were all associated with higher prevalence of AD in both univariable and multivariable GLM adjusted by age (all p<0.05). DM and depression were all associated with higher CRP, while carrying *APOE* E4 was associated with lower CRP levels in both univariable and multivariable GLM (all p< 0.001) in all populations.

**Conclusions:**

DR and depression, as comorbidities related to blood-retinal barrier and blood-brain barrier impairment in patients with DM, may play pivotal roles in the development of POAG and AD among DM patients.

## Introduction

Glaucoma, affecting over 60 million people globally, is the second leading cause of blindness, with POAG being predominant. Glaucoma is characterized by the progressive loss of retinal ganglion cells (RGCs) through apoptosis (1, 2), which shares molecular similarities with central nervous system (CNS) degenerative disorders like AD, indicating a common pathological mechanism. The retina, an extension of the CNS, and its barrier (inner blood-retinal barrier, iBRB) resemble the blood-brain barrier (BBB) (3). It was reported that circulating immune cell migration through an impaired BBB and glial activation contributes to the progression of AD (4). Similarly, impairment of the iBRB is observed in glaucoma, which has been proven to be pivotal in determining the fate or prognosis of neuroinflammation pathological outcomes for POAG (5). A current theory proposes that when systemic immune and inflammatory components entering the retina or brain through the impaired iBRB or BBB initiate a self-exacerbating cycle of neuroimmune responses, then it would lead to the development of clinical disease phenotypes such as POAG or AD (6). Similar pattern has been stated in previous studies about brain structural changes and neurodegenerative processes in relation to clinical severity and cognitive symptoms in glaucoma (7–10). Although, there is little agreement on the role of chronic systemic diseases in developing of neurodegenerative diseases (11, 12), this novel perspective could enhance our understanding of the etiology, mechanisms, and potential therapies for POAG and AD.

DM, a common systemic disease, is associated with heightened risks of both POAG and AD (13, 14). DR and depression are common comorbidities of DM (15, 16), sharing pathological mechanisms that contribute to the breakdown of iBRB and BBB, potentially playing a causative role in POAG and AD (17). Associations have been demonstrated in previous studies, including several meta-analyses (18, 19), between POAG with AD (20, 21), DM and depression (22). Therefore, we hypothesized that DR and depression, serving as indicators of iBRB and BBB impairment, might act as intermediary processes between DM and POAG and AD, and could possibly accelerate the progression of POAG and AD. Meanwhile, these processes were also interfered with intrinsic factors associated with AD and POAG, such as age, gender and *APOE* E4 status (23). A coherent pathological picture that explains these complex relationships is likely to describe molecular and mechanistic similarities of these disorders and may pave the way for the development of novel and effective therapies. This study aims to develop a comprehensive model exploring the association between DM and POAG, AD, across different gender groups and *APOE* E4 genotypes in a large cohort, in which depression and DR serve as intermediate factors, with CRP levels evaluated to gauge inflammatory conditions in different contexts.

## Materials and methods

### Ethics statement

UKBB received approval from the North West Multi-centre Research Ethics Committee. Recruitment for the UKBB was obtained by written consent. We have full access to de-identified data with permission approved by UKBB as complying with their Access Procedures and Ethics. Our research adheres to the tenets of the Declaration of Helsinki.

### Study population

The UKBB is a large-scale prospective cohort study of participants recruited from 2006 through 2010 from across the United Kingdom. POAG, AD, depression, DM, and DR phenotypes were identified through data coding based on the International Classification of Diseases, Tenth Revision (ICD10). Out of the entire UKBB cohort including 502,505 participants, the final sample consisted of 28112 participants with DM and 471869 participants without DM considered as controls after excluding patients lacking DM information. We retrieved visual acuity from both eyes (data fields: 5208 and 5201). The best recorded visual acuity from either eye at the two time points was converted to its logMAR equivalent and used as visual acuity (VA) for subsequent analysis. Circulating CRP levels were measured using high-sensitivity assays [data-field 30710, initial assessment visit (2006–2010)].

### Genotyping

DNA microarray genotyping was generated using Axiom arrays (including the UKBB Lung Exome Variant Evaluation (BiLEVE) and UKBB arrays; Thermo Fisher) for the UKBB. *APOE* alleles (E1, E2, E3, and E4) were determined from 2 relevant Single Nucleotide Polymorphisms (SNPs) within the *APOE* gene (rs429358 and rs7412; GRCh38 reference genome) (24). Because of the rarity of the E1 allele, rare E1 genotypes (E1E2 and E1E4) were excluded from the analysis. Apolipoprotein SNPs were measured directly from Axiom arrays. Because the relevant *APOE* SNPs were not included on the Illumina arrays, they were imputed in Minimac3 using Haplotype Reference Consortium r1.1 as a reference panel (rs429358 imputation R2 ¼ 0.93; rs7412 imputation R2 ¼ 0.92). *APOE* E4 carriers was classified as carrying ≥1 copy (E24 + E34 + E44).

### Statistics

Statistical analyses were conducted using R (version 4.2.1) with publicly available packages. Demographic parameters were initially compared through chi-square tests, and independent sample t-tests. The univariable generalized linear model (GLM) was used to analyze factors related to POAG, AD, and CRP levels, adjusting for age (the one recorded at the time of recruitment in this cohort and was utilized in subsequent analysis) in both genders and each gender. Multivariate GLMs were further conducted, incorporating DM/DR, *APOE* E4 status and depression as variables, adjusting for age and gender. For the allelic regression, an indicator variable for E4 status, defined by the presence or absence of the relevant allele, was included. In this context, the β_e4_ represents the impact of the E4 allele, indicating a value of 1 or 0 to represent its presence or absence, respectively. Since depression was more likely to be affected by the awareness of the illness and its effect on patients’ daily life, we further explored the association between depression and POAG, DM, DR using GLM, adjusting for age, sex, and VA. Analysis of variance (ANOVA) was used to compare the differences among Models. The threshold for statistical significance (α) was set at p<0.05.

## Results

### Population

A total of 28,112 patients with DM and 471,869 without DM were included in the study. The DM group was further divided into two subcategories: 2,097 patients with DR and 9,932 with DM but without DR. A final classification round was done for depression status, with 1,874 patients having both depression and DM, and 26,238 with DM but without depression.

### Comparing characteristics between patients with and without DM/depression and between DM patients with and without DR/depression

The comparisons of the demographics and prevalence of depression, AD, and POAG between patients with and without DM and between DM patients with and without DR were presented in **Table 1**. Older age, more males, and worse VA were presented in patients with DM compared with patients without DM and in DM patients with DR compared with those without DR (all p<0.001). Depression, AD, and POAG were more prevalent in participants with DM compared with non-DM participants, and if DM patients had DR, the prevalence of those comorbidities was even higher than those without DR (all p<0.05). CRP was increased in DM patients (2.54 ± 4.30 vs. 3.49 ± 5.15mg/L; p<0.001), and even higher in those with DR (3.40 ± 4.80 vs. 3.79 ± 5.74mg/L; p<0.001). Though DM patients had a higher percentage of carrying *APOE* E4 (28.60% vs.26.77%; p<0.001), the distribution of *APOE* E4 allele status did not differ significantly in DM patients with or without DR (p=0.729) (**Table 1**).

**Table 1:** Comparison depression, POAG, AD, APOE E4 status and level of CRP in patients with and without DM or DR and in female and male patients with DM or DR.

Subjects	No DM	DM	P	No DR with DM	With DR	p	Female DM	Male DM	P	Female DR	Male DR	p
n	471869	28112	<0.001^††^	9932	2097	<0.001^††^	11008	17104	<0.001^††^	748	1349	<0.001^††^
Age	56.36 ± 8.11	59.41 ± 7.24	<0.001^††^	59.12 ± 7.26	60.12 ± 7.18	<0.001^††^	58.72 ± 7.49	59.84 ± 7.05	<0.001^††^	59.61 ± 7.27	60.41 ± 7.11	<0.001^††^
Gender (male%)	44.64	60.84	<0.001^††^	5912 (59.52)	1349 (64.33)	<0.001^††^	–	–	–	–	–	–
VA (logMAR)	-0.05 ± 0.15	-0.02± 0.16	<0.001^††^	-0.03 ± 0.15	0.07 ± 0.19	<0.001^††^	-0.01 ± 0.16	-0.02 ± 0.16	<0.001^††^	0.09 ± 0.19	0.06 ± 0.18	0.077
Depression (n,%)	17045 (3.61)	1874 (6.67)	<0.001^††^	596 (6.00)	245 (11.68)	<0.001^††^	62 (0.56)	131 (0.77)	0.053	9 (1.20)	26 (1.93)	0.288
AD (n,%)	879 (0.19)	117 (0.42)	<0.001^††^	23 (0.23)	23 (1.10)	<0.001^††^	891 (8.09)	983 (5.75)	<0.001^††^	114 (15.24)	131 (9.71)	<0.001^††^
POAG (n,%)	1664 (0.35)	193 (0.69)	<0.001^††^	91 (0.92)	35 (1.67)	0.003^†^	46 (0.42)	71 (0.42)	1	10 (1.34)	13 (0.96)	0.571
*APOE* E4 carriers^*^ (n,%)	131245 (28.60)	7273 (26.77)	<0.001^††^	2541 (26.51)	541 (26.91)	0.729	2863 (27.11)	4410 (26.56)	0.319	200 (28.21)	341 (26.21)	0.362
CRP (mg/L)	2.54 ± 4.30	3.49 ± 5.15	<0.001^††^	3.40 ± 4.80	3.79 ± 5.74	0.005^†^	4.31 ± 5.67	2.97 ± 4.72	<0.001^††^	5.60 ± 6.60	3.10 ± 5.07	<0.001^††^

^*^APOE E4 carriers:carrying ≥1 copy (E24 + E34 + E44);

DM, diabetes mellitus; POAG, primary open angle glaucoma; AD, Alzheimer’s disease; DR, diabetic retinopathy. CRP, C-reactive protein; VA, visual acuity.

^†^P<0.05; ^††^P<0.001.

**Table 2** demonstrated the demographics and prevalence of depression, AD, and POAG between patients with and without depression and between DM patients with and without depression. Younger age, more females, and worse VA were presented in patients with depression compared with patients without depression and in DM patients with depression compared with those without depression (All p<0.001). DM, DR, AD, and POAG were more prevalent in participants with depression compared with non-depression participants, and if DM patients had depression, the prevalence of DR and AD were even higher than those without depression (all p<0.05), while the prevalence of POAG was similar (P=0.854). CRP was increased in depression patients (2.55 ± 4.31 vs. 3.65 ± 5.42mg/L; p<0.001), and even higher in DM patients with depression compared with DM patients without depression (3.40 ± 5.08 vs. 4.70 ± 5.95mg/L; p<0.001). The distribution of *APOE* E4 allele status did not differ significantly in patients with or without depression and in DM patients with or without depression (both p>0.05) (**Table 2**).

**Table 2:** Comparison DM, DR, POAG, AD, APOE E4 status and level of CRP in patients with and without depression and/or DM and the gender differences.

Subjects	No Depression	Depression	P	No Depression with DM	Depression with DM	p	Female depression	Male depression	P	Female Depression with DM	Male Depression with DM	p
n	481062	18919	<0.001^††^	26238	1874	<0.001^††^	11899	7020	<0.001^††^	891	983	0.036^†^
Age	56.55 ± 8.09	56.19 ± 8.05	<0.001^††^	59.53 ± 7.20	57.64 ± 7.58	<0.001^††^	55.92 ± 8.03	56.64 ± 8.05	<0.001^††^	56.98 ± 7.61	58.25 ± 7.50	<0.001^††^
Gender(male%)	45.89	37.11	<0.001^††^	61.44	52.45	<0.001^††^	–	–	–	–	–	–
VA (logMAR)	0.05 ± 0.15	0.02 ± 0.16	<0.001^††^	0.05 ± 0.15	0.02 ± 0.16	<0.001^††^	0.02 ± 0.17	-0.03 ± 0.16	<0.001^††^	0.02 ± 0.18	0.01 ± 0.17	0.395
DM (n,%)	26238(5.45)	1874(9.91)	<0.001^††^	–	–	–	891(7.49)	983(14.00)	<0.001^††^	–	–	–
DR (n,%)	3629(1.80)	363(4.5)	<0.001^††^	3591(27.15)	361(35.92)	<0.001^††^	159(3.14)	204(6.84)	<0.001^††^	159 (33.06)	202(38.55)	0.081
AD (n,%)	843(0.18)	153(0.81)	<0.001^††^	97(0.37)	20(1.07)	<0.001^††^	84(0.71)	69(0.98)	0.049^†^	9(1.01)	11(1.12)	0.997
POAG (n,%)	1752(0.36)	105(0.55)	<0.001^††^	179(0.68)	14(0.75)	0.854	67(0.56)	38(0.54)	0.926	9(1.01)	5(0.51)	0.322
*APOE* E4 carriers^*^ (n,%)	133216(27.69)	5302(29.21)	0.095	6778(26.71)	495 (26.61)	0.415	3295(28.77)	2007(29.53)	0.281	235(27.65)	260(29.48)	1
CRP (mg/L)	2.55 ± 4.31	3.65 ± 5.42	<0.001^††^	3.40 ± 5.08	4.70 ± 5.95	<0.001^††^	3.81 ± 5.34	3.36 ± 5.54	<0.001^††^	5.45 ± 6.03	4.03 ± 5.79	<0.001^††^

^*^APOE E4 carriers:carrying ≥1 copy (E24 + E34 + E44);

DM, diabetes mellitus; POAG, primary open angle glaucoma; AD, Alzheimer’s disease; DR, diabetic retinopathy. CRP, C-reactive protein; VA, visual acuity.

^†^P < 0.05; ^††^P<0.001.

### Comparing characteristics between females and males in patients with DM or DR or depression

The comparisons of the demographics and prevalence of depression, AD, and POAG between female and male patients with DM or DR were presented in **Table 1**. In both DM and DR patients, females had younger age, higher CRP levels, and higher prevalence of AD compared with males (all p<0.001). VA in female patients with DM was worse compared with male patients with DM (p<0.001), there was no difference in VA between females and males with DR (p=0.077). No difference in *APOE* E4 status and the prevalence of depression and POAG between females and males in both DM and DR patients (all p>0.05).

The comparisons of the demographics and prevalence of DM, DR, AD, and POAG between female and male patients with depression were presented in **Table 2**. In depression patients, females had younger age, worse VA, higher CRP levels, and lower prevalence of DR and AD compared with males (all p<0.001). No difference in *APOE* E4 status and the prevalence of POAG between females and males in depression patients (both p>0.05). In DM patients with depression, females had younger age and higher CRP levels (both p<0.001), and no differences were detected in VA, *APOE* E4 status, and the prevalence of DR, AD, and POAG (all p>0.05) between females and males.

### Factors associated with the risk of POAG and AD in patients with and without DM/depression and between DM patients with and without DR/depression

**Table 3** displayed factors associated with the risk of POAG and AD in patients with/without DM. In the univariate and multivariate GLM (Model 1) adjusted by age, male, DM, and depression were all associated with higher prevalence of POAG and AD (all p<0.05), while carrying *APOE* E4 is a protective factor for prevalence of POAG with borderline significance (univariable GLM: OR=0.898, p=0.046; multivariate GLM: OR=0.899, p=0.047) but significant risk factor for prevalence of AD (univariate GLM: OR=4.279, p<0.001; multivariate GLM: OR=4.271, p<0.001). In DM patients, being male or having DR were associated with higher prevalence of POAG in the univariate GLM after adjusting for age (both p<0.05). *APOE* E4 status and depression were not associated with POAG (both p>0.05). In the multivariate GLM (Model 2) after adjusting by age, DR was still associated with higher prevalence of POAG (OR=1.826, p=0.003), while sex, *APOE* E4 status and depression were not associated with the prevalence of POAG. DR, depression and *APOE* E4 positive were associated with higher prevalence of AD both in the univariate and multivariate GLM (Model 2) adjusted by age (all p<0.001). However, sex was not associated with AD in DM patients (p=0.324) (**Table 3**).

**Table 3:** Factors associated with POAG, AD and blood CRP level in participants with/without DM and in DM patients with/without DR (general linear model, adjust for age).

Outcomes	POAG			AD			CRP	
Participants with/without DM	OR	β Coeffificient	P	OR	β Coeffificient	P	β Coeffificient	P
sex (0:female, 1:male)	1.354		< 0.001^††^	1.140		0.039^†^	-0.250 ± 0.013	< 0.001^††^
DM (0/1: without/with)	1.510		< 0.001^††^	1.587		< 0.001^††^	0.856 ± 0.028	< 0.001^††^
*APOE* E4^*^ (0/1: without/with)	0.898		0.046^†^	4.279		< 0.001^††^	-0.622 ± 0.014	< 0.001^††^
depression (0/1: without/with)	1.607		< 0.001^††^	4.946		< 0.001^††^	1.106 ± 0.034	< 0.001^††^
CRP		0.003 ± 0.005	0.947		-0.009 ± 0.008	0.251		
Model 1: DM+APOE+sex+depression								
DM (0/1: without/with)	1.488		< 0.001^††^	1.556		< 0.001^††^	0.853 ± 0.028	< 0.001^††^
*APOE* E4^*^ (0/1: without/with)	0.899		0.047^†^	4.271		< 0.001^††^	-0.619 ± 0.014	< 0.001 ^††^
Sex (0: female, 1: male)	1.354		< 0.001^††^	1.155		0.029^†^	-0.263 ± 0.013	< 0.001 ^††^
Depression (0/1: without/with)	1.307		< 0.001^††^	4.978		< 0.001^††^	0.969 ± 0.035	< 0.001 ^††^
DM patients with/without DR	OR	β Coeffificient	P	OR	β Coeffificient	P	β Coeffificient	P
Sex (0: female, 1: male)	1.517		0.036^†^	0.745		0.324	-1.372 ± 0.096	< 0.001 ^††^
DR (0/1: without/with)	1.691		0.009^†^	4.171		< 0.001^††^	0.421 ± 0.125	< 0.001 ^††^
*APOE* E4^*^ (0/1: without/with)	1.011		0.961	2.615		0.023^†^	-0.697 ± 0.107	< 0.001 ^††^
depression (0/1: without/with)	1.207		0.591	4.321		< 0.001^††^	1.498 ± 0.188	< 0.001 ^††^
CRP		0.017 ± 0.015	0.256		0.012 ± 0.027	0.660		
Model 2: DR+APOE+sex+depression								
DR (0/1: without/with)	1.826		0.003^†^	3.994		< 0.001^††^	0.382 ± 0.124	0.002 ^†^
*APOE* E4^*^ (0/1: without/with)	1.011		0.957	1.836		< 0.001^††^	-0.729 ± 0.105	< 0.001^††^
Sex (0: female, 1: male)	1.396		0.101	0.731		0.287	-1.329 ± 0.096	< 0.001 ^††^
Depression (0/1: without/with)	1.095		0.807	3.139		< 0.001^††^	1.270 ± 0.192	< 0.001 ^††^
DM patients with/without depression	OR	β Coeffificient	P	OR	β Coeffificient	P	β Coeffificient	P
Sex (0: female, 1: male)	1.265		0.130	0.882		0.508	-1.309 ± 0.065	< 0.001^††^
DR (0/1: without/with)	1.452		0.025 ^†^	3.072		< 0.001^††^	0.152 ± 0.098	0.121
*APOE* E4^*^ (0/1: without/with)	0.891		0.502	2.702		< 0.001^††^	-0.801 ± 0.072	< 0.001^††^
depression (0/1: without/with)	1.261		0.407	3.699		< 0.001^††^	1.243 ± 0.129	< 0.001^††^
CRP		0.001 ± 0.015	0.955		-0.012 ± 0.021	0.589		
Model 3: DR+APOE+sex+depression								
DR (0/1: without/with)	1.530		0.001 ^††^	3.053		< 0.001^††^	0.160 ± 0.096	0.096
*APOE* E4^*^ (0/1: without/with)	0.980		0.913	1.963		0.015 ^†^	-0.769 ± 0.097	< 0.001^††^
Sex (0: female, 1: male)	1.265		0.184	0.650		0.114	-1.244 ± 0.089	< 0.001^††^
Depression (0/1: without/with)	1.212		0.544	3.701		< 0.001^††^	1.403 ± 0.170	< 0.001^††^

^*^APOE E4 status: 0: non-carriers = E22 + E23 + E33; 1: carriers = E24 + E34 + E44;

DM, diabetes mellitus; POAG, primary open angle glaucoma; AD, Alzheimer’s disease; DR, diabetic retinopathy. CRP, C-reactive protein.

^†^P < 0.05; ^††^P<0.001.

Being female, having DM and experiencing depression were all associated with higher CRP, while carrying *APOE* E4 was associated with lower CRP in both univariate and multivariate GLM (all p< 0.001) in all populations (all participants with/without DM and DM patients with/without DR or depression, **Table 3**). CRP was not associated with either POAG or AD in all populations (all p>0.05).

**Figure 1:**
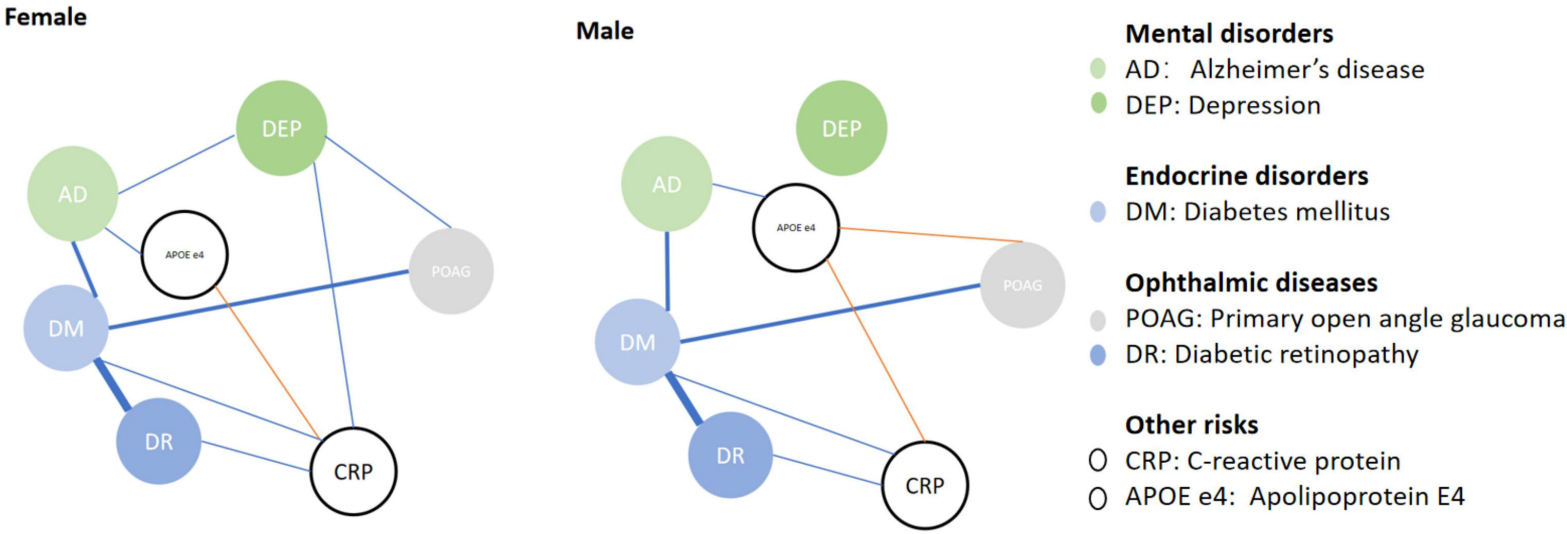
Diagram comparing health condition networks between females and males.

We further analyzed factors associated with POAG, AD and blood CRP levels in different genders (**Table 4**) and have the association visualized in **Figure 1**. After adjusting for age, DM was associated with higher prevalence of POAG in both genders (female, OR_POAG_=1.504, p<0.001; male, OR_POAG_=1.417, p<0.001). *APOE* E4 was not associated with POAG in females (p=0.433), while it was associated with a lower prevalence of POAG in males (OR=0.863, p=0.045). Only among female patients, depression was associated with a higher prevalence of POAG (OR_POAG_=1.980, p<0.001). DM and depression were still associated with a higher prevalence of POAG in females in multivariate GLM adjusted by age (model 4, both p<0.05). While DM and not carrying *APOE* E4 status were associated with a higher prevalence of POAG in males in multivariate GLM adjusted by age (model 4, both p<0.05). In both genders, DM, carrying *APOE* E4 and depression were all associated with a higher prevalence of AD in both univariate and multivariate GLM adjusted by age (all p<0.05).

**Table 4:** Factors associated with POAG, AD and blood CRP level in female and male participants with/without DM (general linear model, adjust for age).

Outcomes	POAG		AD		CRP	
Female	OR	P	OR	P	β Coeffificient	P
DM (0/1: without/with)	1.504	0.002^†^	1.824	<0.001^††^	1.593 ± 0.044	<0.001^††^
*APOE* E4^*^ (0/1: without/with)	0.940	0.438	5.218	<0.001^††^	-0.613 ± 0.019	<0.001^††^
depression (0/1: without/with)	1.980	<0.001^††^	4.885	<0.001 ^††^	1.174 ± 0.042	<0.001 ^††^
CRP	1.006	0.374	1.000	0.987		
Model 4: DM+*APOE* E4+depression						
DM (0/1: without/with)	1.496	0.003^†^	1.824	<0.001^††^	0.853 ± 0.028	<0.001^††^
*APOE* E4^*^ (0/1:without/with)	0.934	0.433	5.199	<0.001^††^	-0.619 ± 0.014	<0.001^††^
Depression (0/1: without/with)	1.980	<0.001^††^	4.749	<0.001 ^††^	0.969 ± 0.035	<0.001^††^
Male	OR	P	OR	P	β Coeffificient	P
DM (0/1: without/with)	1.417	<0.001^††^	1.419	0.007^†^	0.445 ± 0.036	<0.001^††^
*APOE* E4^*^ (0/1: without/with)	0.863	0.045^†^	3.569	<0.001^††^	-0.631 ± 0.021	<0.001^††^
depression (0/1: without/with)	1.251	0.177	5.181	<0.001^††^	0.928 ± 0.055	<0.001^††^
CRP	0.996	0.618	1.000	0.9871		
Model 5: DM+*APOE* E4+depression						
DM (0/1: without/with)	1.496	<0.001^††^	1.406	0.010^†^	0.407 ± 0.036	<0.001^††^
*APOE* E4^*^ (0/1: without/with)	0.940	0.049^†^	3.570	<0.001^††^	-0.630 ± 0.021	<0.001^††^
Depression (0/1: without/with)	1.995	0.250	5.244	<0.001 ^††^	0.906 ± 0.055	<0.001^††^

^*^APOE E4 status: 0: non-carriers = E22 + E23 + E33; 1: carriers = E24 + E34 + E44;

DM, diabetes mellitus; POAG, primary open angle glaucoma; AD, Alzheimer’s disease; DR, diabetic retinopathy. CRP, C-reactive protein.

^†^P < 0.05;^††^P < 0.001.

DM and depression were all associated with higher CRP, while carrying *APOE* E4 was associated with lower CRP level in both univariate and multivariate GLM (all p< 0.001) in both female and male population. CRP level was not associated with either POAG or AD in both female and male populations (all p>0.05).

### Factors associated with depression in patients with/without DM

**Table 5** displayed factors associated with depression in patients with/without DM. In the univariable GLM adjusted by age, factors like POAG, DM, female, and worse VA were all associated with higher prevalence of depression (OR_POAG_=1.580, p<0.001; OR_DM_=1.952, p<0.001; OR_Gender_=0.697, p<0.001; β_VA_=1.087 ± 0.086, p<0.001), while carrying *APOE* E4 was not associated with depression(p=0.100). After adjusting the VA and sex, POAG showed no association with depression (p=0.131). Both DM and DR showed an association with higher prevalence of depression after adjusting the VA and sex (OR_DM_=2.066, p<0.001; OR_DR_=2.420, p<0.001).

**Table 5:** Factors associated with depression in patients with/without DM (general linear model, adjust for age).

Outcomes	Depression		
Factors	OR	β Coeffificient	P
Sex (0: female, 1: male)	0.697		<0.001^††^
DM (0/1: without/with)	1.952		<0.001^††^
DR in DM (0/1: without/with)	2.164		<0.001^††^
*APOE* E4 ^*^(0/1: without/with)	1.028		0.100
VA (logMAR)		1.087 ± 0.086	<0.001^††^
POAG	1.580		<0.001^††^
Model 1: POAG+VA+sex			
POAG	1.405		0.131
VA (logMAR)		1.039 ± 0.087	<0.001^††^
Sex (0: female, 1: male)	0.733		<0.001^††^
Model 2: DM+VA+sex			
Sex (0: female, 1: male)	0.707		<0.001^††^
DM (0/1: without/with)	2.066		<0.001^††^
VA (logMAR)		0.993 ± 0.088	<0.001^††^
Model 3: DR+VA+sex			
Sex (0: female, 1: male)	0.772		0.013^†^
DR (0/1: without/with)	2.420		<0.001^††^
VA (logMAR)		1.045 ± 0.304	<0.001^††^

^*^APOE E4 status: 0: non-carriers = E22 + E23 + E33; 1: carriers = E24 + E34 + E44;

DM, diabetes mellitus; POAG, primary open angle glaucoma; AD, Alzheimer’s disease; DR, diabetic retinopathy. CRP, C-reactive protein; VA, visual acuity.

^†^P < 0.05; ^††^P<0.001.

## Discussion

DM was found to be associated with heightened risks of both POAG and AD in previous studies, likely due to shared pathological mechanisms such as oxidative stress, inflammation, vascular dysfunction, and impaired insulin signaling pathways (25). Thus, our study supports a current hypothesis that POAG and AD may be thought of as diabetes of the brain (25). Additionally, immune and inflammatory components from the systemic circulation enter the brain and retina through the impaired BBB and BRB, which are also implicated in DM-related DR and depression, and could also initiate a self-exacerbating vicious cycle of neuroimmune responses that lead to the development of POAG and AD (6). In this study, we first explored the associations between DM and POAG, AD, on the conception that DR and depression serve as intermediate factors between these associations. We discovered that patients with DR showed higher prevalence of POAG, together with higher prevalence of AD among DM patients with DR and depression. Moreover, we tried to integrate the effects of *APOE* E4 allele, gender and CRP into our models to enhance our understanding of the common pathological mechanisms in neurodegeneration and further explain the associations among DM, POAG, and AD (26).

Consistent with our findings, an Indian study identified a positive association between glaucoma and DR in a type 2 diabetes mellitus (T2DM) population (OR=2.62) (27). Additionally, other studies have shown that T2DM patients with POAG have a threefold higher risk of developing DR compared to those without POAG (28). Similarly, research using the Danish Registry of Diabetic Retinopathy found that patients with DM and either glaucoma or ocular hypertension were more likely to develop DR within five years (29). Clinical diagnosis of both POAG and DR are often abrupt, making early detection and monitoring of progression challenging. Whilst these findings do not establish causality, they reinforce the association between POAG and DR. Indeed, we continue to regard the development of DR as a crucial factor in the progression of POAG, particularly concerning the role of the BRB in glaucoma. Normally, the BRB’s integrity limits retinal damage; minor disruptions in the BRB are typically transient and quickly repaired, preventing clinical consequences (5). Thus, age-related BRB breakdown alone may not cause POAG. Histological studies have shown BRB damage in glaucoma models, with abnormalities in retinal pigment epithelium (RPE) cells, increased permeability of retinal vessels, and leukocyte infiltration (2). In animal models of transient high intraocular pressure (IOP) glaucoma, BRB impairment has been linked to T-cell infiltration and progressive ganglion cell death (30, 31). These findings suggested that BRB integrity is crucial in POAG progression and breakdown of BRB was the essential factor in the very early stages of POAG (5, 31). DM, especially DR, can compromise the BRB, leading to neuroinflammation in the retina, which may contribute to glaucoma progression. Early dysfunction of the neurovascular unit (NVU) in diabetes has been observed in animal models and patients, leading to impaired neurovascular coupling, loss of autoregulation, and disruption of the iBRB (32). Factors like hypoxia-ischemia, oxidative stress, and inflammation during DR development contribute to both inner and outer BRB breakdown (18, 33–35). These processes indicate that BRB impairments could even precede the clinical onset of DR. The relationship between POAG severity and a significant decrease in retinal vessel density at ONH and macula level further suggests that microvascular damage in the diabetic retina exacerbates retinal neurodegeneration (36, 37). Persistent microvascular leakage in advanced DR induces chronic neural immune-inflammatory responses, eventually leading to neuronal loss (38). Therefore, the disruption of the iBRB caused by DM might explain the significant association between POAG and DM. The higher prevalence of POAG among DR patients could be due to a longer duration of DM and more severe BRB and microvascular damage. Although the precise timing and relationship between glial activation, BRB impairment, and immune-inflammatory cell infiltration in POAG need further clarification, comprehensive research could shed light on the mechanisms underlying progressive RGC loss in glaucoma associated with age-related systemic diseases like DM and DR,which may also explain the progressive RGC loss in glaucoma patients with well-controlled or normal IOP, where BRB impairment, triggered by high IOP or other systemic factors, is likely a critical factor leading to ongoing neuroinflammation and RGC loss independent of IOP levels.

Meanwhile, our study has corroborated the association between DM and AD. Numerous investigations have shown a higher incidence of cognitive decline in individuals with DM. Longitudinal studies conducted in Japan (39) and a five-year prospective study by Yaffe et al. (40) have robustly established DM as a risk factor for AD and cognitive decline, particularly in the context of metabolic syndrome. Neurovascular changes in the brain, similar to those seen in the BRB in DM patients, are believed to contribute to the higher risk of AD (41). The retina, an extension of the CNS, shares similarities with the BBB, suggesting that DM-related BBB impairment, particularly in patients with DR, could be expected (3, 42, 43). The BBB, composed of endothelial cells, pericytes, and astrocytes, regulates the passage of substances into the brain and protects against harmful signals from the bloodstream (44). Migration of circulating immune cells through an impaired BBB, along with glial activation, contributes to the progression of AD and may explain the increased prevalence observed in DM patients, particularly those with DR. Moreover, depression, which can be induced by DM through the strain of diabetes itself and changes in the hypothalamus–pituitary–adrenal (HPA) axis or BBB structure (45–47), has been identified as a risk factor for AD and is prevalent in DM patients. Chronic social stress has been shown to alter BBB integrity in animal models, promoting behaviors akin to depression (48, 49). This suggests that BBB dysfunction may play a role in the development of depression and could contribute to the association between DM, DR, and depression. Furthermore, BBB damage may exacerbate the impact of depression on AD, similar to the key role of BRB disruption in DR on the progression of POAG. Additionally, the *APOE* E4 allele, a known risk factor for AD, may contribute to BBB breakdown and neuroinflammation, synergizing with systemic inflammation to promote AD onset (50). Our multivariable GLM analysis, adjusted for age and stratified by sex, demonstrated the complex interplay between DM, depression, *APOE* E4, and AD, underscoring their roles in the pathogenesis of AD.

Although we did not observe an association between POAG and depression after adjusting for age, sex, and VA, previous studies have indicated a higher prevalence of depression in individuals with POAG (22), and increased risk of glaucoma in patients with major depressive disorder (MDD). In our study, females were more likely to experience depression than males, regardless of DM or DR status. Notably, depression was associated with POAG only in females, possibly due to irregular estrogen levels caused by ovarian hormone fluctuations in patients with depression, since estrogen is essential for maintaining RGCs (51, 52). Additionally, we observed a protective effect of the *APOE* E4 allele in males against POAG, even with DM, which aligns with previous research indicating a reduced risk of POAG associated with this allele (53). Similar findings were revealed by previous studies which showed a difference of AD prevalence among different gender (54). Interestingly, the *APOE* E4 allele is a known risk factor for AD, suggesting a mechanistic difference between neurodegenerative diseases affecting the eye and brain. This difference may be due to *APOE* E4 acting as an example of antagonistic pleiotropy—a gene that provides benefits at one stage of life but later presents disadvantages (55)—since POAG typically manifests at a younger age than AD. The post-menopausal loss of estrogen was key in the increased incidence of AD in women (56). Considering the accelerated aging process post-menopause, particularly in females with DM and depression (57–60), the protective effect of the *APOE* E4 allele may be diminished in this group. Both clinical researches (61, 62) and transgenic mice model (63, 64) has revealed that combination of *APOE4* genotype and female sex often exacerbated outcomes across numerous cognitive loss. Although further studies are needed to clarify these mechanisms, these gender differences highlight the complex interplay of DM, depression, and *APOE* E4 in the context of POAG. Additionally, the correlation between retinal vascular abnormalities, cognitive impairment, and dementia, as well as the similarity between the BRB and the BBB, suggests that retinal function may serve as a promising biomarker for neurodegenerative diseases (65). Previous clinical studies have already shown that POAG patients are at a higher risk of developing AD compared to controls (66) and vice versa (67). Therefore, considering the earlier and more accessible detection of POAG compared to AD, we propose that POAG could potentially be used as an early screening indicator for AD in patients with DM.

Furthermore, although we attempted to elucidate the connection between DM, POAG, and AD by assessing inflammation levels, represented by blood CRP levels, and hypothesized that proinflammatory cytokine levels might explain breaches in the BRB and BBB, our study did not find any association between blood CRP levels and POAG or AD. Despite observing increased CRP levels in DM, which further increased in patients with DR and depression, particularly among females, no correlation with POAG or AD was identified. We further tried to integrate the effects of *APOE* E4 allele, gender and CRP into our models to enhance our understanding of the common pathological mechanisms in neurodegeneration and further explain the associations among DM, POAG, and AD (26). CRP is a marker of systemic inflammation and increases with age. Although multiple AD-related genes are associated with the level of CRP, the association between blood CRP levels and risk of AD are not conclusive in the literature, with studies showing both low and high levels of CRP in patients with AD (68). Similarly, peripheral inflammatory markers have been linked to a higher risk of vascular dementia, but not AD (31). A Mendelian randomization study suggested a protective effect of CRP on AD, possibly influenced by the methods used to measure CRP (69). It was reported that in the context influenced by possessing the *APOE* E4 allele, variations in CRP levels—either transformation or suppression—may lead to reduced blood CRP levels (70, 71). Previous studies have observed an inverse correlation between *APOE* E4 and blood CRP levels, but only within the lower CRP level range, across both elderly and younger populations (72–74). In a large UK Biobank subsample with elevated CRP levels, it was shown that the effect of genetic variants on CRP diminished as CRP levels increased (75). This suggests that chronic, low-grade inflammation, as indicated by CRP, is strongly influenced by genetic factors such as *APOE* E4, rather than reflecting CRP’s response to acute infections or other stimuli. This may explain why *APOE* E4 carriers in our study exhibited lower CRP levels regardless of gender or diabetes status, and why we found no association between CRP levels and AD or POAG. The complex inflammatory profiles involving *APOE* E4, AD, DM, and POAG may offer insights into the systemic inflammation underlying these distinct but related neurodegenerative processes. Future research investigating other proinflammatory cytokines, such as interleukin-6 (IL-6), and exploring CRP’s role in modulating genetic risk for POAG and AD, may further elucidate these complex interactions (76).

Although the underlying mechanisms still require further investigation, previous clinical studies and our data analysis have come to similar conclusion that POAG might come from the consequence of a primary neurodegenerative disease of the CNS, together with BRB and BBB breakdown (77). This insight opens up potential treatment pathways that focus on neuroprotective molecules rather than IOP-targeted medications for POAG. Currently, Coenzyme Q1 (CoQ10) and citicoline are among the most commonly used neuroprotective agents in POAG treatment. CoQ10, a crucial antioxidant that safeguards proteins and DNA from oxidative stress, has been shown to prevent optic nerve astrocyte activation induced by hydrogen peroxide *in vitro* while also inhibiting RGC apoptosis and loss in animal models (78). Clinical trials and animal studies further suggest that CoQ10 protects optic nerve head (ONH) astrocytes from oxidative stress primarily by preserving mitochondrial function (79). Similarly, citicoline has demonstrated neuroprotective properties by reducing glutamate-mediated excitotoxicity and oxidative stress through the enhancement of neurotrophin levels and mitochondrial support (78). Preliminary studies indicate that the combination of citicoline and vitamin B12 eye drops can help stabilize neuroretinal degeneration and mitigate microvascular damage in DR patients (80). Given these findings, the combined use of CoQ10 and citicoline presents a promising new strategy for glaucoma treatment. Overall, these emerging therapeutic approaches for POAG further support our findings.

Several limitations were identified in our study, particularly our inability to pinpoint the exact onset times of DM, DR, depression, POAG, and AD. Determining the precise onset of each disease is challenging because symptoms may not manifest in early stages, and recorded onset times often only reflect the age at diagnosis. This limitation has prevented us from fully elucidating the causal relationships and could only presented as possible connection between these conditions. Also, we used ICD-10 codes for ascertaining clinical phenotypes might capture a heterogeneous group of patients. An ideal study sample would include a large, population-based cohort with comprehensive ocular phenotyping and chronic disease phenotypes. However, clinical criteria for diagnosis vary in sensitivity across different diseases, which would still impact the consistency and reliability of our findings. Secondly, in this study, we only included age, sex, CRP, and *APOE* E4 as variables. However, there are undoubtedly additional variables that could influence the complex interplay among DM, POAG, and AD. We believed our results were still valuable for understanding the shared pathological mechanisms involved in DM, POAG, and AD. Thirdly, although European ancestry represented the predominant ethnic group within this study cohort, ethnic variations in glaucoma prevalence and *APOE* E4 allele distribution were not addressed, limiting analysis to diverse ethnic groups. Including more diverse populations in future analyses could strengthen the findings and enhance their applicability to global populations.

## Conclusions

In summary, our findings suggested that DR and depression, as comorbidities related to BRB and BBB impairment in patients with DM, may play crucial roles in the development of POAG and AD among DM patients. Although the complexities of these interactions require further detailed characterization, they provide valuable insights into the underlying mechanisms and potential shared pathways in the development of POAG, AD, and other neurodegenerative diseases. Enhancing knowledge and awareness of these associations could lead to the development of new avenues in the understanding and management of glaucoma, AD, and DM.

## Data Availability

Publicly available datasets were analyzed in this study. This data can be found here: https://biobank.ndph.ox.ac.uk/showcase/search.cgi.

## References

[1] Qu J Wang D Grosskreutz CL. Mechanisms of retinal ganglion cell injury and defense in glaucoma. Exp Eye Res. (2010) 91:48–53. doi: 10.1016/j.exer.2010.04.002, PMID: 20394744 PMC3378677

[2] Okisaka S Murakami A Mizukawa A Ito J. Apoptosis in retinal ganglion cell decrease in human glaucomatous eyes. Jpn J Ophthalmol. (1997) 41:84–8. doi: 10.1016/s0021-5155(97)00013-0, PMID: 9152810

[3] Ohtsuki S Yamaguchi H Katsukura Y Asashima T Terasaki T. mRNA expression levels of tight junction protein genes in mouse brain capillary endothelial cells highly purified by magnetic cell sorting. J Neurochem. (2008) 104:147–54. doi: 10.1111/j.1471-4159.2007.05008.x, PMID: 17971126

[4] Mietelska-Porowska A Wojda U. T lymphocytes and inflammatory mediators in the interplay between brain and blood in alzheimer's disease: potential pools of new biomarkers. J Immunol Res. (2017) 2017:4626540. doi: 10.1155/2017/4626540, PMID: 28293644 PMC5331319

[5] Shi X Li P Herb M Liu H Wang M Wang X. Pathological high intraocular pressure induces glial cell reactive proliferation contributing to neuroinflammation of the blood-retinal barrier via the NOX2/ET-1 axis-controlled ERK1/2 pathway. J Neuroinflammation. (2024) 21:105. doi: 10.1186/s12974-024-03075-x, PMID: 38649885 PMC11034147

[6] Yang X Yu XW Zhang DD Fan ZG. Blood-retinal barrier as a converging pivot in understanding the initiation and development of retinal diseases. Chin Med J (Engl). (2020) 133:2586–94. doi: 10.1097/CM9.0000000000001015, PMID: 32852382 PMC7722606

[7] Minosse S Garaci F Martucci A Lanzafame S Di Giuliano F Picchi E. Primary open angle glaucoma is associated with functional brain network reorganization. Front Neurol. (2019) 10:1134. doi: 10.3389/fneur.2019.01134, PMID: 31708862 PMC6823877

[8] Mancino R Cesareo M Martucci A Di Carlo E Ciuffoletti E Giannini C. Neurodegenerative process linking the eye and the brain. Curr Med Chem. (2019) 26:3754–63. doi: 10.2174/0929867325666180307114332, PMID: 29521197

[9] Di Cio F Garaci F Minosse S Passamonti L Martucci A Lanzafame S. Reorganization of the structural connectome in primary open angle Glaucoma. NeuroImage Clin. (2020) 28:102419. doi: 10.1016/j.nicl.2020.102419, PMID: 33032067 PMC7552094

[10] Martucci A Cesareo M Toschi N Garaci F Bagetta G Nucci C. Brain networks reorganization and functional disability in glaucoma. Prog Brain Res. (2020) 257:65–76. doi: 10.1016/bs.pbr.2020.07.007, PMID: 32988473

[11] Yin J Li H Guo N. Prevalence of depression and anxiety disorders in patients with glaucoma: A systematic review and meta-analysis based on cross-sectional surveys. Actas Esp Psiquiatr. (2024) 52:325–33. doi: 10.62641/aep.v52i3.1561, PMID: 38863056 PMC11190447

[12] Kim HK Lee W Ryu IH Kim JK Kim H Yoo TK. Association between metformin use and the risk of developing open-angle glaucoma among patients with diabetes: a retrospective cohort study and meta-analysis. Int Ophthalmol. (2024) 44:6. doi: 10.1007/s10792-024-02945-w, PMID: 38316664

[13] Law SK Hosseini H Saidi E Nassiri N Neelakanta G Giaconi JA. Long-term outcomes of primary trabeculectomy in diabetic patients with primary open angle glaucoma. Br J Ophthalmol. (2013) 97:561–6. doi: 10.1136/bjophthalmol-2012-302227, PMID: 23355527

[14] Diniz Pereira J Gomes Fraga V Morais Santos AL Carvalho MDG Caramelli P Braga Gomes K. Alzheimer's disease and type 2 diabetes mellitus: A systematic review of proteomic studies. J Neurochem. (2021) 156:753–76. doi: 10.1111/jnc.15166, PMID: 32909269

[15] Song P Yu J Chan KY Theodoratou E Rudan I. Prevalence, risk factors and burden of diabetic retinopathy in China: a systematic review and meta-analysis. J Glob Health. (2018) 8:10803. doi: 10.7189/jogh.08.010803, PMID: 29899983 PMC5997368

[16] Cooper DH Ramachandra R Ceban F Di Vincenzo JD Rhee TG Mansur RB. Glucagon-like peptide 1 (GLP-1) receptor agonists as a protective factor for incident depression in patients with diabetes mellitus: A systematic review. J Psychiatr Res. (2023) 164:80–9. doi: 10.1016/j.jpsychires.2023.05.041, PMID: 37331261

[17] Harerimana NV Liu Y Gerasimov ES Duong D Beach TG Reiman EM. Genetic evidence supporting a causal role of depression in alzheimer’s disease. Biol Psychiatry. (2022) 92:25–33. doi: 10.1016/j.biopsych.2021.11.025, PMID: 35177243 PMC9200901

[18] Zhao YX Chen XW. Diabetes and risk of glaucoma: systematic review and a Meta-analysis of prospective cohort studies. Int J Ophthalmol. (2017) 10:1430–5. doi: 10.18240/ijo.2017.09.16, PMID: 28944204 PMC5596230

[19] Zhou M Wang W Huang W Zhang X. Diabetes mellitus as a risk factor for open-angle glaucoma: a systematic review and meta-analysis. PloS One. (2014) 9:e102972. doi: 10.1371/journal.pone.0102972, PMID: 25137059 PMC4138056

[20] Della Santina L Inman DM Lupien CB Horner PJ Wong RO. Differential progression of structural and functional alterations in distinct retinal ganglion cell types in a mouse model of glaucoma. J Neurosci. (2013) 33:17444–57. doi: 10.1523/JNEUROSCI.5461-12.2013, PMID: 24174678 PMC3812509

[21] Poon WW Blurton-Jones M Tu CH Feinberg LM Chabrier MA Harris JW. beta-Amyloid impairs axonal BDNF retrograde trafficking. Neurobiol Aging. (2011) 32:821–33. doi: 10.1016/j.neurobiolaging.2009.05.012, PMID: 19540623 PMC3038182

[22] Liu CH Kang EY Lin YH Wu WC Liu ZH Kuo CF. Association of ocular diseases with schizophrenia, bipolar disorder, and major depressive disorder: a retrospective case-control, population-based study. BMC Psychiatry. (2020) 20:486. doi: 10.1186/s12888-020-02881-w, PMID: 33008365 PMC7532110

[23] Ravipati K Chen Y Manns JR. Reassessing diabetes and APOE genotype as potential interacting risk factors for alzheimer's disease. Am J Alzheimers Dis Other Demen. (2022) 37:15333175211070912. doi: 10.1177/15333175211070912, PMID: 35041557 PMC10623968

[24] Tisato V Zuliani G Vigliano M Longo G Franchini E Secchiero P. Gene-gene interactions among coding genes of iron-homeostasis proteins and APOE-alleles in cognitive impairment diseases. PloS One. (2018) 13:e0193867. doi: 10.1371/journal.pone.0193867, PMID: 29518107 PMC5843269

[25] Nguyen TT Ta QTH Nguyen TKO Nguyen TTD Giau VV. Type 3 diabetes and its role implications in alzheimer's disease. Int J Mol Sci. (2020) 21:3165. doi: 10.3390/ijms21093165, PMID: 32365816 PMC7246646

[26] Gemmati D Longo G Gallo I Silva JA Secchiero P Zauli G. Host genetics impact on SARS-CoV-2 vaccine-induced immunoglobulin levels and dynamics: The role of TP53, ABO, APOE, ACE2, HLA-A, and CRP genes. Front Genet. (2022) 13:1028081. doi: 10.3389/fgene.2022.1028081, PMID: 36531241 PMC9748098

[27] Behera UC Bhattacharjee H Das T Gilbert C Murthy GVS Rajalakshmi R. group Ss. Spectrum of Eye Disease in Diabetes (SPEED) in India: A prospective facility-based study. Report 4. Glaucoma in people with type 2 diabetes mellitus. Indian J Ophthalmol. (2020) 68:S32–6. doi: 10.4103/ijo.IJO_1948_19, PMID: 31937726 PMC7001170

[28] Abikoye TM Oluleye TS Aribaba OT Musa KO Idowu OO Onakoya AO. Is primary open-angle glaucoma a risk factor for diabetic retinopathy? Int Ophthalmol. (2020) 40:3233–40. doi: 10.1007/s10792-020-01507-0, PMID: 32696101

[29] Sperling S Stokholm L Thykjaer AS Pedersen FN Moller S Laugesen CS. Bidirectional 5-year risks of diabetic retinopathy, glaucoma and/or ocular hypertension: Results from a national screening programme. Acta Ophthalmol. (2023) 101:384–91. doi: 10.1111/aos.15300, PMID: 36514165

[30] Xu H Manivannan A Liversidge J Sharp PF Forrester JV Crane IJ. Requirements for passage of T lymphocytes across non-inflamed retinal microvessels. J Neuroimmunol. (2003) 142:47–57. doi: 10.1016/s0165-5728(03)00258-3, PMID: 14512163

[31] Ravaglia G Forti P Maioli F Chiappelli M Montesi F Tumini E. Blood inflammatory markers and risk of dementia: The Conselice Study of Brain Aging. Neurobiol Aging. (2007) 28:1810–20. doi: 10.1016/j.neurobiolaging.2006.08.012, PMID: 17011077

[32] Nguyen TT Kawasaki R Kreis AJ Wang JJ Shaw J Vilser W. Correlation of light-flicker-induced retinal vasodilation and retinal vascular caliber measurements in diabetes. Invest Ophthalmol Vis Sci. (2009) 50:5609–13. doi: 10.1167/iovs.09-3442, PMID: 19643973

[33] Huber JD VanGilder RL Houser KA. Streptozotocin-induced diabetes progressively increases blood-brain barrier permeability in specific brain regions in rats. Am J Physiol Heart Circ Physiol. (2006) 291:H2660–8. doi: 10.1152/ajpheart.00489.2006, PMID: 16951046

[34] Starr JM Wardlaw J Ferguson K MacLullich A Deary IJ Marshall I. Increased blood-brain barrier permeability in type II diabetes demonstrated by gadolinium magnetic resonance imaging. J Neurol Neurosurg Psychiatry. (2003) 74:70–6. doi: 10.1136/jnnp.74.1.70, PMID: 12486269 PMC1738177

[35] Acharya NK Levin EC Clifford PM Han M Tourtellotte R Chamberlain D. Diabetes and hypercholesterolemia increase blood-brain barrier permeability and brain amyloid deposition: beneficial effects of the LpPLA2 inhibitor darapladib. J Alzheimers Dis. (2013) 35:179–98. doi: 10.3233/JAD-122254, PMID: 23388174

[36] Ramm L Jentsch S Peters S Sauer L Augsten R Hammer M. Dependence of diameters and oxygen saturation of retinal vessels on visual field damage and age in primary open-angle glaucoma. Acta Ophthalmol. (2016) 94:276–81. doi: 10.1111/aos.12727, PMID: 25876673

[37] Martucci A Giannini C Di Marino M Sorge RP Aiello F Scuteri D. Evaluation of putative differences in vessel density and flow area in normal tension and high-pressure glaucoma using OCT-angiography. Prog Brain Res. (2020) 257:85–98. doi: 10.1016/bs.pbr.2020.07.006, PMID: 32988475

[38] Joussen AM Poulaki V Le ML Koizumi K Esser C Janicki H. A central role for inflammation in the pathogenesis of diabetic retinopathy. FASEB J. (2004) 18:1450–2. doi: 10.1096/fj.03-1476fje, PMID: 15231732

[39] Ohara T Doi Y Ninomiya T Hirakawa Y Hata J Iwaki T. Glucose tolerance status and risk of dementia in the community: the Hisayama study. Neurology. (2011) 77:1126–34. doi: 10.1212/WNL.0b013e31822f0435, PMID: 21931106

[40] Yaffe K Kanaya A Lindquist K Simonsick EM Harris T Shorr RI. The metabolic syndrome, inflammation, and risk of cognitive decline. JAMA. (2004) 292:2237–42. doi: 10.1001/jama.292.18.2237, PMID: 15536110

[41] Kopf D Frolich L. Risk of incident Alzheimer's disease in diabetic patients: a systematic review of prospective trials. J Alzheimers Dis. (2009) 16:677–85. doi: 10.3233/JAD-2009-1011, PMID: 19387104

[42] Nitta T Hata M Gotoh S Seo Y Sasaki H Hashimoto N. Size-selective loosening of the blood-brain barrier in claudin-5-deficient mice. J Cell Biol. (2003) 161:653–60. doi: 10.1083/jcb.200302070, PMID: 12743111 PMC2172943

[43] Argaw AT Gurfein BT Zhang Y Zameer A John GR. VEGF-mediated disruption of endothelial CLN-5 promotes blood-brain barrier breakdown. Proc Natl Acad Sci U S A. (2009) 106:1977–82. doi: 10.1073/pnas.0808698106, PMID: 19174516 PMC2644149

[44] Segarra M Aburto MR Acker-Palmer A. Blood-brain barrier dynamics to maintain brain homeostasis. Trends Neurosci. (2021) 44:393–405. doi: 10.1016/j.tins.2020.12.002, PMID: 33423792

[45] Hasan SS Clavarino AM Dingle K Mamun AA Kairuz T. Diabetes mellitus and the risk of depressive and anxiety disorders in Australian women: A longitudinal study. J Womens Health (Larchmt). (2015) 24:889–98. doi: 10.1089/jwh.2015.5210, PMID: 26121486

[46] Arshad AR Alvi KY. Frequency of depression in type 2 diabetes mellitus and an analysis of predictive factors. J Pak Med Assoc. (2016) 66:425–9., PMID: 27122269

[47] Dunlavey CJ. Introduction to the hypothalamic-pituitary-adrenal axis: healthy and dysregulated stress responses, developmental stress and neurodegeneration. J Undergrad Neurosci Educ. (2018) 16:R59–60., PMID: 30057514 PMC6057754

[48] Menard C Pfau ML Hodes GE Kana V Wang VX Bouchard S. Social stress induces neurovascular pathology promoting depression. Nat Neurosci. (2017) 20:1752–60. doi: 10.1038/s41593-017-0010-3, PMID: 29184215 PMC5726568

[49] Dudek KA Dion-Albert L Lebel M LeClair K Labrecque S Tuck E. Molecular adaptations of the blood-brain barrier promote stress resilience vs. depression. Proc Natl Acad Sci U S A. (2020) 117:3326–36. doi: 10.1073/pnas.1914655117, PMID: 31974313 PMC7022213

[50] Zhou X Shi Q Zhang X Gu L Li J Quan S. ApoE4-mediated blood-brain barrier damage in Alzheimer's disease: Progress and prospects. Brain Res Bull. (2023) 199:110670. doi: 10.1016/j.brainresbull.2023.110670, PMID: 37224887

[51] Fotesko K Thomsen BSV Kolko M Vohra R. Girl power in glaucoma: the role of estrogen in primary open angle glaucoma. Cell Mol Neurobiol. (2022) 42:41–57. doi: 10.1007/s10571-020-00965-5, PMID: 33040237 PMC11441221

[52] Albert KM Newhouse PA. Estrogen, stress, and depression: cognitive and biological interactions. Annu Rev Clin Psychol. (2019) 15:399–423. doi: 10.1146/annurev-clinpsy-050718-095557, PMID: 30786242 PMC9673602

[53] Margeta MA Letcher SM Igo RP Jr. Cooke Bailey JN Pasquale LR Haines JL. Association of APOE with primary open-angle glaucoma suggests a protective effect for APOE epsilon4. Invest Ophthalmol Vis Sci. (2020) 61:3. doi: 10.1167/iovs.61.8.3, PMID: 32614373 PMC7425753

[54] Belloy ME Andrews SJ Le Guen Y Cuccaro M Farrer LA Napolioni V. APOE genotype and alzheimer disease risk across age, sex, and population ancestry. JAMA Neurol. (2023) 80:1284–94. doi: 10.1001/jamaneurol.2023.3599, PMID: 37930705 PMC10628838

[55] Riedel BC Thompson PM Brinton RD. Age, APOE and sex: Triad of risk of Alzheimer's disease. J Steroid Biochem Mol Biol. (2016) 160:134–47. doi: 10.1016/j.jsbmb.2016.03.012, PMID: 26969397 PMC4905558

[56] Paganini-Hill A Henderson VW. Estrogen replacement therapy and risk of Alzheimer disease. Arch Intern Med. (1996) 156:2213–7. doi: 10.1001/archinte.1996.00440180075009 8885820

[57] Poehlman ET Tchernof A. Traversing the menopause: changes in energy expenditure and body composition. Coron Artery Dis. (1998) 9:799–803. doi: 10.1097/00019501-199809120-00004, PMID: 9894924

[58] Kara A Unal D Simsek N Yucel A Yucel N Selli J. Ultra-structural changes and apoptotic activity in cerebellum of post-menopausal-diabetic rats: a histochemical and ultra-structural study. Gynecol Endocrinol. (2014) 30:226–31. doi: 10.3109/09513590.2013.864270, PMID: 24397360

[59] Herson M Kulkarni J. Hormonal agents for the treatment of depression associated with the menopause. Drugs Aging. (2022) 39:607–18. doi: 10.1007/s40266-022-00962-x, PMID: 35908135 PMC9355926

[60] An SY Kim Y Kwon R Lim GY Choi HR Namgoung S. Depressive symptoms and suicidality by menopausal stages among middle-aged Korean women. Epidemiol Psychiatr Sci. (2022) 31:e60. doi: 10.1017/S2045796022000439, PMID: 36017644 PMC9428901

[61] Neu SC Pa J Kukull W Beekly D Kuzma A Gangadharan P. Apolipoprotein E genotype and sex risk factors for alzheimer disease: A meta-analysis. JAMA Neurol. (2017) 74:1178–89. doi: 10.1001/jamaneurol.2017.2188, PMID: 28846757 PMC5759346

[62] Hohman TJ Dumitrescu L Barnes LL Thambisetty M Beecham G Kunkle B. Alzheimer's disease genetics C, the alzheimer's disease neuroimaging I. Sex-specific association of apolipoprotein E with cerebrospinal fluid levels of tau. JAMA Neurol. (2018) 75:989–98. doi: 10.1001/jamaneurol.2018.0821, PMID: 29801024 PMC6142927

[63] Nam KN Wolfe CM Fitz NF Letronne F Castranio EL Mounier A. Integrated approach reveals diet, APOE genotype and sex affect immune response in APP mice. Biochim Biophys Acta Mol Basis Dis. (2018) 1864:152–61. doi: 10.1016/j.bbadis.2017.10.018, PMID: 29038051 PMC5714325

[64] Maldonado Weng J Parikh I Naqib A York J Green SJ Estus S. Synergistic effects of APOE and sex on the gut microbiome of young EFAD transgenic mice. Mol Neurodegener. (2019) 14:47. doi: 10.1186/s13024-019-0352-2, PMID: 31861986 PMC6923910

[65] Cheung CY Ikram MK Chen C Wong TY. Imaging retina to study dementia and stroke. Prog Retin Eye Res. (2017) 57:89–107. doi: 10.1016/j.preteyeres.2017.01.001, PMID: 28057562

[66] Lin IC Wang YH Wang TJ Wang IJ Shen YD Chi NF. Glaucoma, Alzheimer's disease, and Parkinson's disease: an 8-year population-based follow-up study. PloS One. (2014) 9:e108938. doi: 10.1371/journal.pone.0108938, PMID: 25275530 PMC4183534

[67] Cesareo M Martucci A Ciuffoletti E Mancino R Cerulli A Sorge RP. Association between alzheimer's disease and glaucoma: A study based on heidelberg retinal tomography and frequency doubling technology perimetry. Front Neurosci. (2015) 9:479. doi: 10.3389/fnins.2015.00479, PMID: 26733792 PMC4683203

[68] Tao Q Ang TFA DeCarli C Auerbach SH Devine S Stein TD. Association of chronic low-grade inflammation with risk of alzheimer disease in apoE4 carriers. JAMA Netw Open. (2018) 1:e183597. doi: 10.1001/jamanetworkopen.2018.3597, PMID: 30646251 PMC6324596

[69] Ligthart S Vaez A Vosa U Stathopoulou MG de Vries PS Prins BP. Genome analyses of >200,000 individuals identify 58 loci for chronic inflammation and highlight pathways that link inflammation and complex disorders. Am J Hum Genet. (2018) 103:691–706. doi: 10.1016/j.ajhg.2018.09.009, PMID: 30388399 PMC6218410

[70] Tao Q Alvin Ang TF Akhter-Khan SC Itchapurapu IS Killiany R Zhang X. Impact of C-reactive protein on cognition and alzheimer disease biomarkers in homozygous APOE varepsilon4 carriers. Neurology. (2021) 97:e1243–52. doi: 10.1212/WNL.0000000000012512, PMID: 34266923 PMC8480484

[71] Lima TA Adler AL Minett T Matthews FE Brayne C Marioni RE. C-reactive protein, APOE genotype and longitudinal cognitive change in an older population. Age Ageing. (2014) 43:289–92. doi: 10.1093/ageing/aft193, PMID: 24305621 PMC3927773

[72] Wang Y Grydeland H Roe JM Pan M Magnussen F Amlien IK. Associations of circulating C-reactive proteins, APOE epsilon4, and brain markers for Alzheimer's disease in healthy samples across the lifespan. Brain Behav Immun. (2022) 100:243–53. doi: 10.1016/j.bbi.2021.12.008, PMID: 34920091

[73] Martiskainen H Takalo M Solomon A Stancakova A Marttinen M Natunen T. Decreased plasma C-reactive protein levels in APOE epsilon4 allele carriers. Ann Clin Transl Neurol. (2018) 5:1229–40. doi: 10.1002/acn3.639, PMID: 30349858 PMC6186931

[74] Haan MN Aiello AE West NA Jagust WJ. C-reactive protein and rate of dementia in carriers and non carriers of Apolipoprotein APOE4 genotype. Neurobiol Aging. (2008) 29:1774–82. doi: 10.1016/j.neurobiolaging.2007.04.020, PMID: 17540481 PMC2593150

[75] Dumitrescu L Mahoney ER Mukherjee S Lee ML Bush WS Engelman CD. Genetic variants and functional pathways associated with resilience to Alzheimer's disease. Brain. (2020) 143:2561–75. doi: 10.1093/brain/awaa209, PMID: 32844198 PMC7447518

[76] Huang J Tao Q Ang TFA Farrell J Zhu C Wang Y. The impact of increasing levels of blood C-reactive protein on the inflammatory loci SPI1 and CD33 in Alzheimer's disease. Transl Psychiatry. (2022) 12:523. doi: 10.1038/s41398-022-02281-6, PMID: 36550123 PMC9780312

[77] Nucci C Martucci A Cesareo M Mancino R Russo R Bagetta G. Brain involvement in glaucoma: advanced neuroimaging for understanding and monitoring a new target for therapy. Curr Opin Pharmacol. (2013) 13:128–33. doi: 10.1016/j.coph.2012.08.004, PMID: 22981808

[78] Martucci A Mancino R Cesareo M Pinazo-Duran MD Nucci C. Combined use of coenzyme Q10 and citicoline: A new possibility for patients with glaucoma. Front Med (Lausanne). (2022) 9:1020993. doi: 10.3389/fmed.2022.1020993, PMID: 36590976 PMC9797721

[79] Zhang X Tohari AM Marcheggiani F Zhou X Reilly J Tiano L. Therapeutic potential of co-enzyme Q10 in retinal diseases. Curr Med Chem. (2017) 24:4329–39. doi: 10.2174/0929867324666170801100516, PMID: 28762311

[80] Parravano M Scarinci F Parisi V Giorno P Giannini D Oddone F. Citicoline and vitamin B(12) eye drops in type 1 diabetes: results of a 3-year pilot study evaluating morpho-functional retinal changes. Adv Ther. (2020) 37:1646–63. doi: 10.1007/s12325-020-01284-3, PMID: 32180131 PMC7140741

